# The Effect of Mono and Hybrid Additives of Ceramic Nanoparticles on the Tribological Behavior and Mechanical Characteristics of an Al-Based Composite Matrix Produced by Friction Stir Processing

**DOI:** 10.3390/nano13142148

**Published:** 2023-07-24

**Authors:** Essam B. Moustafa, Mohammed A. Taha

**Affiliations:** 1 Mechanical Engineering Department, Faculty of Engineering, King Abdulaziz University, Jeddah P.O. Box 80204, Saudi Arabia; 2 Solid State Physics Department, National Research Centre, El Buhouth St., Dokki 12622, Egypt; mtahanrc@gmail.com

**Keywords:** wear, nanocomposite, ceramic, mechanical properties, FSP, tribological behavior

## Abstract

Friction stir processing (FSP) is an effective method for incorporating ceramic nanoparticles into metal matrix composites. This study investigated the effects of single and multiple additions of BN, VC, and SiC nanoparticles on the microstructure refinement and tribological behavior of an AA2024 alloy-based nanocomposite matrix fabricated by FSP. The results showed that adding ceramic nanoparticles, either singly or in combination, led to significant refinement of grain structure and improved wear resistance of the AA2024 alloy-based nanocomposite matrix. Additionally, the study found that combining BN, SiC, and VC nanoparticles produced the most effective effects on refining and reducing grain size. The microhardness behavior of the composite surface resulting from the hybrid particles showed a significant improvement, reaching 94% more than the base alloy. Overall, these results indicate that the multiple additions of ceramic nanoparticles by FSP are a promising approach to improve aluminum alloys’ tribological behavior and mechanical properties.

## 1. Introduction

In recent years, there has been growing interest in developing metallic–ceramic composite materials for use in friction stir processing applications due to their superior mechanical properties when compared to conventional metallic alloys [1,2]. In addition, these materials can also be fabricated using low-cost fabrication techniques, such as powder metallurgy and friction stir processing, that can help to reduce the cost of the final products [3]. Other approaches, like a laser beam and heat processing, may be employed to produce surface composites and improve their microstructure characteristics [4,5]. The nanocomposite metal matrix is one of the important materials employed in current applications and industries. Depending on the nature of use and application, the reinforcing particles of the metal matrix increase the composite’s properties and affect the physical, electrical, and mechanical properties of a metal composite matrix [6,7]. The FSP is a rapid prototyping technique that involves blending or mixing the reinforcement particles with the base metal by rotating the tool at high speed while applying high pressure [8,9]. The main advantages of the FSP over other manufacturing techniques include reduced processing times, improved surface finish quality, reduced energy requirements, and reduced environmental waste [10,11]. FSP is an advanced manufacturing process that offers significant benefits over traditional manufacturing techniques. However, the poor thermal conductivity of ceramics, which can result in thermally induced phase transformations during processing and limit their applicability in high-temperature applications like wear-resistant tools for machining advanced materials, often limits the use of ceramic reinforcements in these materials [12,13,14]. FSP was used to reinforce an AA6061 alloy with hexagonal boron nitride (HBN), and the resulting composite exhibited enhanced surface microhardness and corrosion resistance [15]. The distribution of the reinforcing particles and the surface volume fraction are two additional variables that influence wear resistance.

Many authors report that FSP can significantly refine the grain size of a material, which can lead to improved mechanical properties such as strength, microhardness, and ductility [16]. The grain refinement is caused by the intense plastic deformation that occurs during FSP. This deformation breaks up the large grains in the material and creates a new, finer-grain structure. The finer-grain structure is more resistant to deformation and fracture, which leads to improved mechanical properties [17,18,19]. The utilization of reinforced particles and ceramic materials, including but not limited to SiC, BN, NbC, VC, VC, CWNT, among others [20,21,22,23], has been implemented through the friction stir process (FSP) to enhance and modify the surface characteristics of aluminum subjected to treatment. The FSP gives superior strength, homogeneity, and significant refining microstructure grains to the fabricated composite. SiC and Al_2_O_3_ nanoparticles increased an aluminum alloy’s wear resistance and microhardness [24].

Furthermore, the microstructure refinement significantly impacts the mechanical properties, which can be attributed to the production of equiaxial grains through the FSP process or the inclusion of chemical modifiers. [25]. The excellent dispersion of nanoparticles eliminates fractures and voids, improving strength and toughness, while the smaller grain size increases strength and stiffness [26,27].

FSP produced hybrid nanocomposites with enhanced mechanical properties, increased wear resistance, and microhardness behavior. Hence, the refined grain structure and uniform distribution of reinforcement particles can improve the mechanical properties of the hybrid nanocomposite by increasing the strength, microhardness, and toughness [28]. The strong metallurgical bond between the reinforcement particles and the matrix can further improve the mechanical properties by preventing the reinforcement particles from debonding from the matrix during loading [29]. A friction stir-processed aluminum AA2024 nanocomposite matrix was studied for wear and tribology. Three composites were made with 0%, 10%, and 30% Al_2_O_3_ particles. The composites’ tensile strength, microhardness, and wear resistance increase when aluminum nanoparticles are added to the alloy [30]. The coefficient of friction can be significantly affected by the presence of ceramic reinforcement particles. The size and concentration of the particles can either increase or decrease the coefficient of friction. Studies have shown that increasing the concentration of ceramic particles typically increases the coefficient of friction [31]. The rationale behind this phenomenon is that, with an increase in the concentration of ceramic reinforcement particles, there is a corresponding increase in the number of contact points between the two surfaces, resulting in elevated friction levels.

Additionally, as the size of ceramic reinforcement particles increases, the coefficient of friction is also believed to increase. This can be attributed to larger particles having more surface area, which increases the likelihood of interaction and adhesion between surfaces [32].

The tribological behavior of an Al-Si-Mg/SiC composite manufactured using stir-casting is investigated by [33]; hence, the authors reported that the composites’ wear rate decreased as the volume percentage of SiC increased from 2% to 15%. The Al-Si-Mg alloy had a 0.022 mm^3^/m wear rate, but the composite containing 15% SiC exhibited a 0.004 mm^3^/m wear rate. The addition of tertiary ceramic additives to Al2618+Si_3_N_4_-B4C-Gr hybrid composites can be a promising approach for improving their microhardness and wear characteristics. This could make these composites more suitable for use in automotive applications, where high wear resistance and toughness are required [34].

The improvement of wear resistance and mechanical properties continues to concern many researchers with the presence of many new composite materials. Hence, balancing these characteristics makes it difficult to determine what specific mechanism we can implement to manufacture composites that combine all these features we want in the new materials. Previous research has not used a controlled process to ensure a homogenous distribution of nanoparticles within the matrix structure of the composites. This study uses a controlled process to ensure a homogenous distribution of nanoparticles, which is important for improving the properties of the composites.

Previous research has not provided insights into the mechanism of enhancing these reinforcement particles for the aluminum alloy composite surface. This study provides insights into the mechanism of enhancing these reinforcement particles, which is important for understanding how to improve the properties of the composites.

Therefore, in the current investigation, the reinforcement of AA2024 wrought alloy with single and multiple particles is achieved to study which is more effective in the reinforcement process. This research aims to investigate the effect of SiC, BN, and VC reinforcement nanoparticles on the microhardness, mechanical properties, and wear behavior of single and hybrid composite matrix AA2024 aluminum alloys using friction stir processing under controlled conditions, to ensure a homogenous distribution of nanoparticles within the matrix structure of the composites. This innovative investigation is helping to explain the mechanism of enhancing these reinforcement particles for the aluminum alloy composite surface. Furthermore, we assess if single or hybrid particles are more effective in enhancing surface composites. However, the percentage of the reinforcement particles is controlled during the fabrication process, and the final composite matrices are different from the theoretical calculation; thus, one of the most important limitations of our research was the lack of control over the ratio and concentration of the reinforcement particles inside the aluminum matrix. In future work, we propose finding ways to control the reinforcement rate within the matrix.

## 2. Materials and Methods

**Figure 1:**
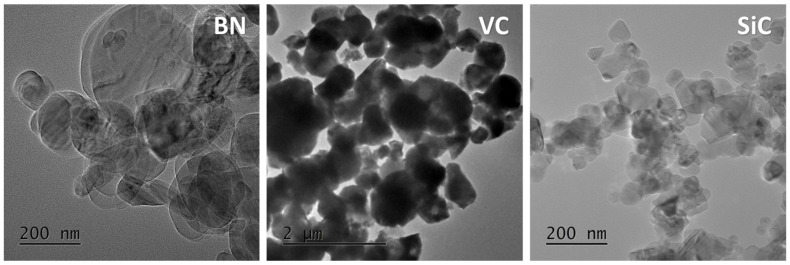
TEM image of the reinforcement particles (BN) boron nitride, (VC) vanadium carbide, and (SiC) silicon carbide powders.

O-annealed AA2024 aluminum alloy was strengthened with SiC and BN nanoparticles, and VC particles to create diverse surface composite matrices. Table 1 summarizes the purities of the reinforcement particles. Table 2 shows the chemical composition of the AA2024 wrought alloy. TEM was used to examine the reinforcement particles’ size (Figure 1). The average particle sizes of VC, SiC, and BN were 1.07 ± 0.4 µm, 30 ± 12 nm, and 140 ± 32 nm, respectively. TEM analysis showed no agglomeration in the powders, and no foreign particles were observed.

### Fabrication Process Using the FSP Method

**Figure 2:**
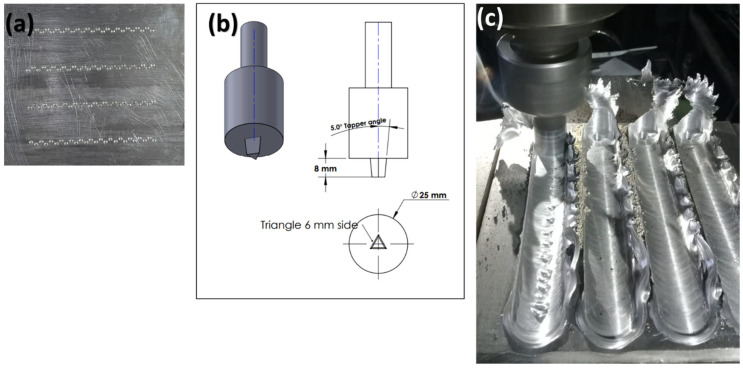
The manufacturing process of the composite surface using the FSP method, (**a**) AA2024 as-received alloy prepared with holes. (**b**) FSP tool design, (**c**) FSP process using a milling machine.

The single and hybrid particles were impeded into the matrix using the friction stir processing (FSP) method, as illustrated in Figure 2, the manufacturing process illustration. The AA2024 aluminum sheets, which have a thickness of 10 mm, exhibit linear holes that have been machined with a diameter of 3 mm and a depth of 3 mm, are created before the FSP (Figure 2a); then, the mono and hybrid particles are immediately inserted into the holes. In the hybrid composite case, the holes were then filled with particles containing equal quantities of the three different reinforcement particles, which were well mixed for excellent distribution and homogeneity. The FSP was performed using a conventional milling machine with a rotation speed of 900 rpm, a processing speed of 30 mm/minute, and a tool tilt angle of 2°. The FSP tool is made from tool steel K110 type; hence, the steel specification is illustrated in Table 3. The tool pin was designed as triangular, as described in (Figure 2b). Samples were cut for characterization after the FSP (microstructure, mechanical, and wear). The AA2024 base alloy and composite samples were polished and etched with standard metallurgical agents for microstructure characterization. Olympus’ BX51 optical microscope and JEOL scanning electron microscopy were used for the microstructure study. The grain size is computed using line intercept theory [35].

*Wear test*: The wear test is performed using pin-on-disk tribometers (CSM Instruments, Peseux, Switzerland), which were utilized to characterize the wear behavior of the FSP tester-fabricated base sample at room temperature. The test was carried out with a standard steady load of 5 N. The moving pin’s radius was 2 mm, and its linear velocity was 0.1 cm/s; the total time was set at 15 min for each test. The coefficient of friction was computed using the normal load ratio and the friction force between the steel pin and the sample. The volumetric wear rate was measured and analyzed using a 3D optical profiler; a non-contact, three-dimensional profilometer built using chromatic confocal technology, which utilizes light wavelengths to calculate physical height precisely.

As a result of relative motion and surface friction, there is a weight reduction known as “wear.” Specific wear rate (*Ws*) is determined by the volume variation of the material after wear (*V*), the normal load applied (*F*), and the sliding distance (*D*), as shown:

$$Ws=\frac{V}{F\times D}$$

*XRD analysis:* The phase composition of the AA2024 alloy and its composites were characterized by the X-ray diffraction (XRD) technique using a “Philips PW 1373.” XRD patterns are used to determine the crystal size and the number of dislocations.

## 3. Results and Discussions

### 3.1. Microstructure Observation

**Figure 3:**
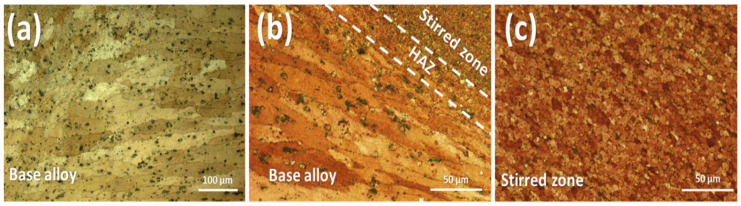
Optical microstructure images of the (**a**) AA2024 base alloy, (**b**) Illustration of the mixed zones formed by FSP process, (**c**) Friction-stirred processed zone in the hybrid composite of AA2024/SiC+BN+VC.

**Figure 4:**
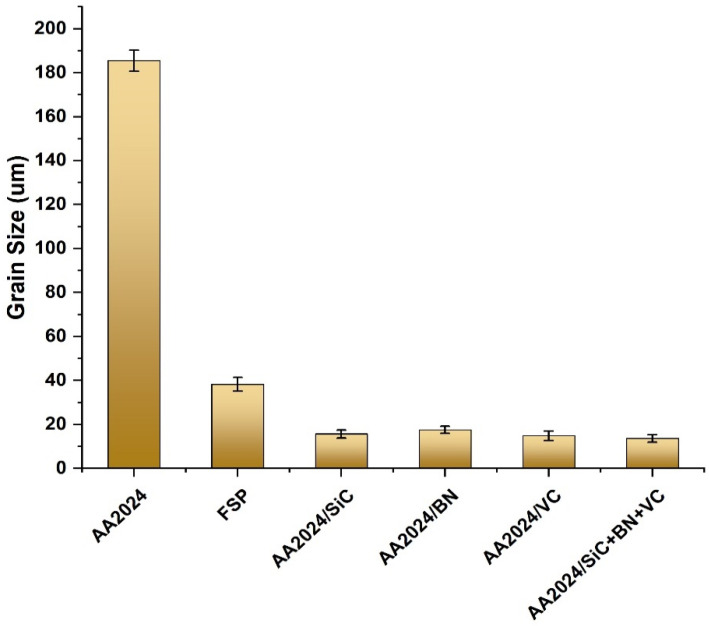
The grain size distribution through the base and composite samples.

The optical microscopic images of the AA2024 alloy sheets in their as-received state and the hybrid composites produced are presented in Figure 3. The microstructure of the as-received alloy is characterized by elongated grains, with an average grain size of 186.5 ± 4.8 µm. In the stirred zone, the created composites have a refined microstructure due to FSP and hybrid reinforcement particles. Due to the semi-solid thermomechanical deformation caused by the FSP, the grains in the stirred zone were completely recrystallized, creating refined equiaxed grains. Owing to the presence of macro/nanoparticles, the Zenner pinning effect increased grain fining by inhibiting grain growth upon recrystallization. The average grain size of the mono-composites of AA2024/SiC, AA2024/BN, and AA2024/VC was 15.6 ± 1.8 µm, 17.5 ± 1.7 µm, and 14.8 ± 2.1 µm, respectively, as shown in Figure 4. In comparison, the average grain size of the hybrid composite for the AA2024/SiC+BN+VC was 13.6 ± 1.73 µm. The type of reinforcing particles can influence grain size by either hindering or promoting grain growth. For example, hard particles can hinder grain growth by providing a barrier to the movement of grain boundaries. This can lead to a smaller grain size, which can improve the composite’s mechanical properties; these results are consistent with [36,37].

Ceramic nanoparticles are essential for reducing grain size during friction stir processing. They pin mobile dislocations, which restricts grain growth. Their high surface area-to-volume ratio allows them to activate the material efficiently during processing, resulting in a refined microstructure. Ceramic nanoparticles also lower the recrystallization temperature and increase nucleation sites in the processed material. This results in a fine and uniform microstructure, improved mechanical properties such as higher strength and ductility, increased wear resistance, and reduced defects.

Scanning electron microscopy (SEM) observation and energy dispersive X-ray spectroscopy (EDX) analysis were used to analyze the reinforcement contents inside the single and hybrid composite to confirm the distribution and composition of nanoparticles in the reinforced aluminum matrix. SEM observation and EDX analysis of the nanoparticles provide valuable information about their morphology, distribution, and composition within the reinforced aluminum matrix.

**Figure 5:**
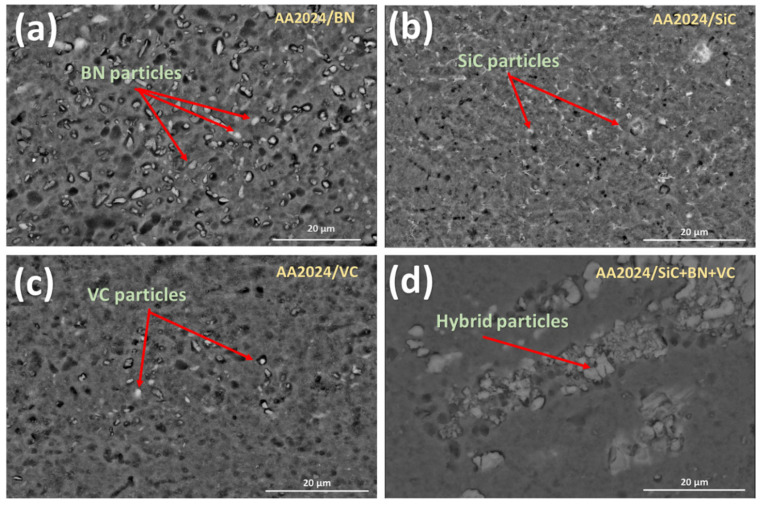
SEM images of the fabricated (**a**) mono-composite AA2024/BN, (**b**) mono-composite AA2024/SiC, (**c**) mono-composite AA2024/VC, (**d**) hybrid composite AA2024/SiC+BN+VC.

SEM images have been used to study the distribution of ceramic nanoparticles within the metal matrix composite (MMC) material after FSP, as shown in Figure 5. These images have shown that SiC, BN, and VC ceramic nanoparticles were uniformly distributed in the metal matrix composite material after FSP. The uniform distribution of ceramic nanoparticles is essential for achieving consistently enhanced mechanical and physical properties throughout the MMC material. The refined microstructure from adding ceramic nanoparticles during FSP also contributes to the overall improvement in mechanical properties and performance, compared to MMCs produced without ceramic nanoparticle additions.

**Figure 6:**
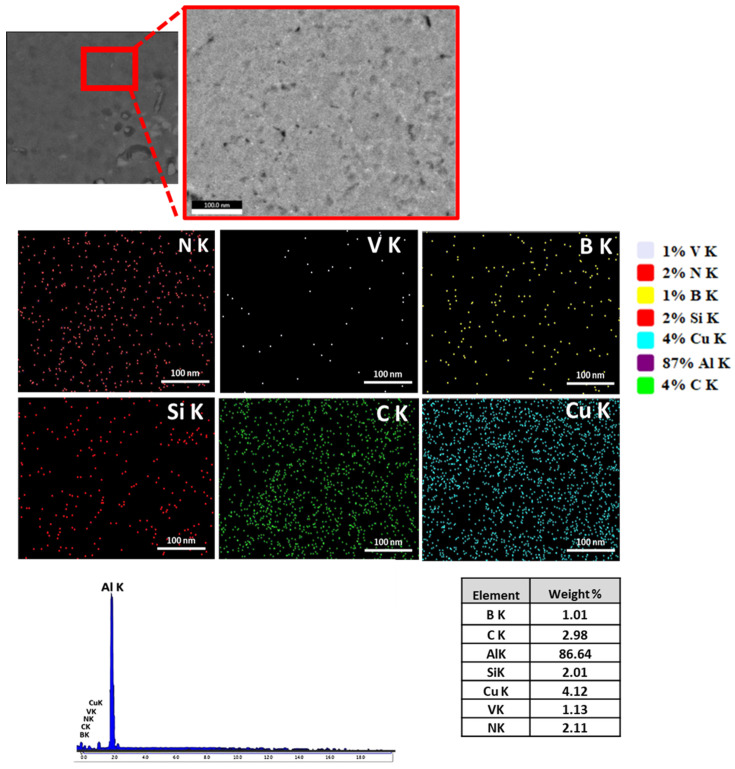
Elemental mapping of the SEM image and EDX analysis for the hybrid AA2024/SiC+BN+VC composite.

However, the addition of multiple ceramic nanoparticles can lead to agglomerated zones within the composite. This is because the different powders have different flow characteristics, and the degree of plasticization that occurs during FSP is not always sufficient to distribute the powders evenly. As a result, the SEM image of the AA2024/SiC+BN+VC hybrid shows a less uniform distribution of reinforcement particles than the other mono-composites. The EDS maps in Figure 6 show that the hybrid composite contains all three types of reinforcement particles, but they are not as evenly distributed as in the mono-composites. This suggests that the addition of multiple ceramic nanoparticles can be beneficial for improving the mechanical properties of MMCs, but it is important to ensure that the powders are distributed evenly to achieve the desired results.

### 3.2. Phase Composition

**Figure 7:**
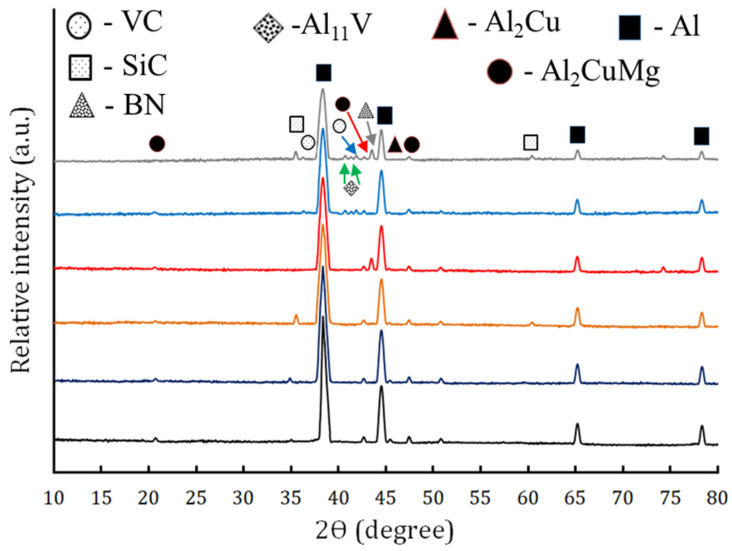
The XRD patterns of hybrid composite surface AA2024/SiC+BN+VC.

The phase composition of the samples was measured by XRD analysis. As observed in Figure 7, for the AA2024 sample, according to (ICCD file card: 44-1288), peaks at 38:43°, 44:68°, 65:07°, and 66:59° are typically used to identify the existence of Al. However, according to card numbers 89-1981 and 28-0014, certain undissolved coarse precipitates dispersed over the base alloy’s grain boundaries were intermetallic phases Al_2_Cu at 47.38, 42.66, and 20.7° and the Al_2_CuMg at 45.33°. According to the previous cards, the Al exhibits a cubic crystal structure, the Al_2_Cu exhibits a cubic crystal structure, and the Al_2_CuMg exhibits a cubic crystal structure. On the other hand, the SiC, BN, and VC ceramic phases also showed up in the AA2024/SiC, AA2024/BN, and AA2024/VC composite samples, respectively, with all ceramic peaks showing up in the hybrid composite, as indicated by card numbers 89-2225, 89-1498, and 74-1220, respectively. It is also noticeable that the AlV phase appears (according to card 07-0281) both in the AA2024/VC and AA2024/SiC+BN+VC samples as a result of the dissolution of part of the VC during the FSP process and the interaction of the V with a simple part of Al. Figure 7 displays the effect of FSP and addition of ceramic on the crystal size and dislocation density of the AA2024 alloy. As is clear from the figure, adding ceramics during the FSP process leads to a decrease in crystal size and an increase in the dislocation density of Al peaks, especially in the sample containing hybrid reinforcement. This may be due to addition of the ceramic leading to an increase in the refinement of the Al grain and, thus, causing a decrease in the Al peaks’ intensity. The dissolution and deposition of reinforcement particles can have a significant impact on the tribological properties of aluminum composites. For example, the dissolution of Al_2_Cu particles can lead to a decrease in hardness and wear resistance, while the deposition of Al_2_Cu particles can lead to an increase in hardness and wear resistance [38]. The formation of new precipitates can also have a significant impact on the tribological properties of aluminum composites. For example, the formation of AlV precipitates can lead to an increase in hardness and wear resistance [39]. The overall effect of precipitate formation on tribological properties depends on the type of precipitates, the processing parameters, and the operating conditions.

The fabricated composites ought to possess a low-weight profile while concurrently enhancing their resistance to wear. Consequently, it is imperative to compute the density of the fabricated hybrid composites and ascertain the ones exhibiting the lowest weight. Equations (1) and (2) are utilized to compute the overall volume of the composite material generated [40].

(1) $$V_{C}=V_{p}+V_{m}$$

(2) $$V_{C}=projected\;area\;of\;tool\times L$$

(3) $$V_{p}=№\;of\;holes\times volume\;of\;each\;hole$$

(4) $$V_{m}=V_{c}-V_{p}$$

(5) $$\rho_{c}=\frac{\left({\rho_{p}\times V}_{p})+{{(\rho }_{m}V}_{m}\right)}{V_{c}}$$

The volume of the composite (*V_c_*), the reinforcement particles (*V_p_*), and the matrix (*V_m_*) are denoted by *V_c_, V_p_*, and *V_m_*, respectively. The length of the manufactured composite is 100 mm in this study, and *L* denotes it. The total volume of the manufactured composite is 4500 mm^3^. The volume of the used nanoceramics and the matrix can be calculated using Equations (3) and (4). The overall theoretical density of the composite can be computed as shown in Equation (5). Table 4 summarizes the various hybrid reinforcing particle ceramics and their theoretical densities in the produced composites.

### 3.3. Microhardness

**Figure 8:**
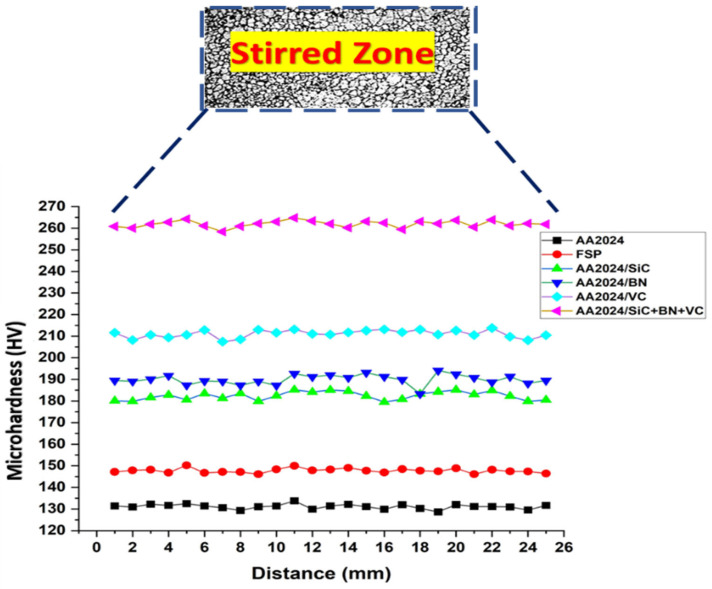
The microhardness behavior of the investigated samples inside the stirred zone.

The microhardness profiles of aluminum surface composites dispersed with single and hybrid reinforcement particle mixtures utilizing FSP inside the stirred zone are illustrated in Figure 8. The data suggest a positive correlation between particle dispersion and microhardness, indicating that an increase in particle dispersion leads to a corresponding increase in microhardness. The type of reinforcing particles can also influence hardness by increasing the resistance of the composite to plastic deformation. This is because the hard particles can act as obstacles to the movement of dislocations, which are the defects that are responsible for plastic deformation [41].

It is clear from the microhardness profile that the hybrid composite showed the highest value for the microhardness inside the stirred zone, while the lowest value is recorded for the AA2024 alloy, followed by the friction-stirred processed alloy. The addition of reinforcement particles such as SiC, BN, and VC to the aluminum matrix resulted in a significant improvement in microhardness. It was observed that the incorporation of single and hybrid composite particles led to a significant increase in surface microhardness, with the hybrid composites demonstrating higher surface microhardness values than single-particle composites. Hence, the AA2024/SiC+BN+VC hybrid composite surface recorded the maximum microhardness value of 260 HV, while the mono-composite AA2024/VC had the second higher value for microhardness with 210 HV. The mono-composite matrix containing BN and SiC nanoparticles had a microhardness value lower than VC particles by 36.5% and 44.44% than the hybrid composite surface. This can be attributed to VC particles having particle sizes more than BN and SiC nanoparticles, which can improve the microhardness behavior of the processed surface. The reinforcement particles appear to enhance the strength of grain boundaries in the composite and restrict dislocation motion. Internal grain boundaries become reinforced, and a dispersion strengthening mechanism occurs due to evenly distributed particles forming throughout the matrix. These findings revealed that the choice of reinforcement particles and their concentration and distribution within the matrix is crucial in determining the microhardness behavior of the composite matrix fabricated through friction stir-processing. The results revealed that adding reinforcement ceramic particles such as SiC, BN, and VC could significantly enhance the microhardness behavior of MMCs fabricated through friction stir processing, making them suitable for use in high-stress and high-temperature applications.

### 3.4. Wear Behavior of the Processed Surface

**Figure 9:**
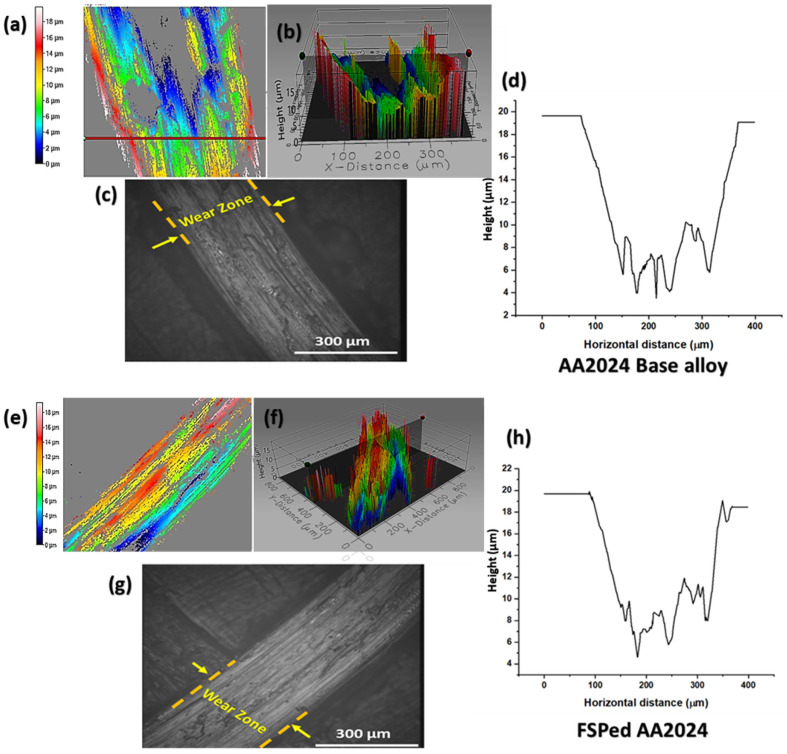
The wear behavior of the as-received AA2024 and friction stir processed AA2024 alloy. (**a**,**e**) Two-dimensional profile of the wear area; (**b**,**f**) the 3D optical image of the wear profile; (**c**,**g**) the optical microscope image of the worn zone; and (**d**,**h**) the wear profile curve captured using a three-dimensional profilometer.

The study of tribological behavior and wear characteristics of materials is of paramount importance in the realm of manufacturing industries. The wear behavior of the aluminum alloy AA2024 is of utmost importance in ensuring its reliability and durability, particularly in its extensive usage in aerospace and automotive domains. A pin-on-disk (POD) test is a tribological test used to measure two materials’ wear and friction properties. In a POD test, a pin made of one material is pressed against a rotating disk made of another material. The POD test conducted on AA2024 and FSP samples revealed the occurrence of adhesive wear. Adhesive wear is a commonly observed type that results from the interaction and adhesion between two surfaces (as illustrated in Figure 9).

**Figure 10:**
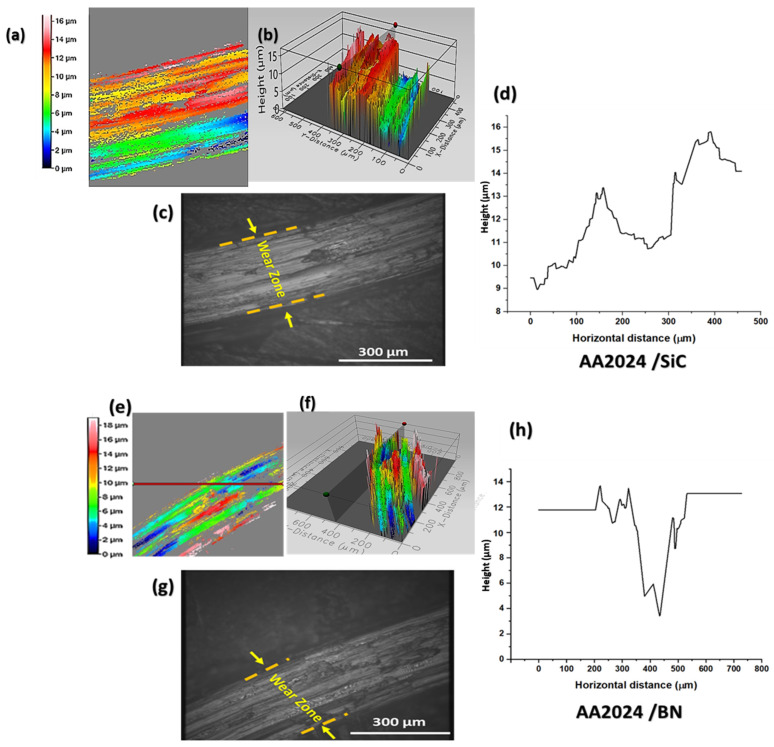
The wear behavior of the mono-composites AA2024/SiC and AA2024/BN. (**a**,**e**) Two-dimensional profile of the wear area; (**b**,**f**) the 3D optical image of the wear profile; (**c**,**g**) the optical microscope image of the worn zone and (**d**,**h**) the wear profile curve.

**Figure 11:**
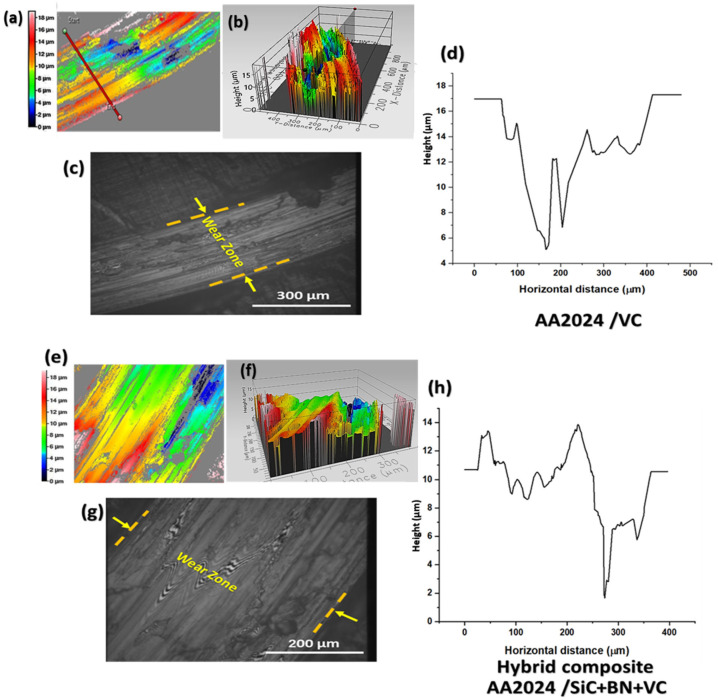
Different wear behaviors are shown by the mono-composite AA2024/VC and the hybrid composite AA2024/SiC+BN+VC. The worn zone’s 2D profile (**a**,**e**); three-dimensional optical picture of the wear profile (**b**,**f**); optical microscope image of the worn zone (**c**,**g**); and wear profile curve (**d**,**h**).

Integrating ceramic reinforcement particles, specifically SiC, BN, and VC, into the metal matrix has positively affected the wear resistance of metal matrix composites (MMCs). Friction stir processing (FSP) has been widely utilized to fabricate metal matrix composites (MMCs) with enhanced wear-resistant properties. As mentioned earlier, the phenomenon can be attributed to the ability of FSP to produce a uniform and finely distributed assortment of strengthening particles. The present research examines the influence of individual and combined reinforcement particles, namely SiC, BN, and VC, on the tribological performance of a composite matrix fabricated using Friction Stir Processing (FSP). The influence of reinforcement particle properties such as type, size, and concentration on the wear behavior of Metal Matrix Composites (MMCs) is a crucial aspect that determines their suitability for use in various industrial contexts that involve wear and abrasion. Silicon carbide (SiC) is a rigid and brittle material commonly used as a reinforcement element in metal matrix composites. Incorporating SiC particles in the AA2024 matrix of a composite material can enhance its wear resistance by augmenting its microhardness and mitigating the frictional forces between the sliding surfaces. The SiC particles function as rigid inclusions that exhibit resistance to deformation and impede the onset of surface wear on the material. Silicon carbide (SiC) particles have the potential to function as lubricants, thereby mitigating frictional forces between sliding surfaces and enhancing their resistance to wear. The AA2024/BN mono-composite comprises boron nitride (BN), a material with notable thermal conductivity and a low coefficient of friction. This renders it a promising option for enhancing wear resistance in materials that undergo high temperatures or loads. Incorporating BN particles into the composite matrix of AA2024 has been observed to mitigate frictional forces between sliding surfaces and impede the onset of surface wear, as depicted in Figure 10. Incorporating VC particles into the AA2024 composite matrix (AA2024/VC) results in a singular composite material exhibiting enhanced wear resistance. This is attributed to the increased microhardness and reduced friction between sliding surfaces that are facilitated by the presence of VC particles. The VC particles function as rigid inclusions that impede the material’s deformation and avert surface wear, as illustrated in Figure 11.

Incorporating hybrid reinforcement particles, namely SiC, BN, and VC, onto the composite surface of the AA2024 aluminum alloy has a notable impact on its tribological and wear characteristics. Incorporating hybrid reinforcement particles into the AA2024/SiC+BN+VC composite resulted in a noteworthy decrease in the friction coefficient between the composite surface and other contacting surfaces, thereby reducing friction. This phenomenon is because said particles function as solid lubricants, diminishing the frictional forces’ magnitude. The improved wear resistance of the composite surface can be attributed to the presence of hybrid reinforcement particles, which reduce the wear rate and enhance the material’s overall durability. The rationale behind this phenomenon is that said particles function as obstructions that impede direct contact between mutually contacting surfaces. In addition, incorporating hybrid particles into surface composites has been observed to augment their capacity to withstand wear by diminishing abrasive and adhesive wear and constraining the generation of microcracks and other forms of impairment.

**Figure 12:**
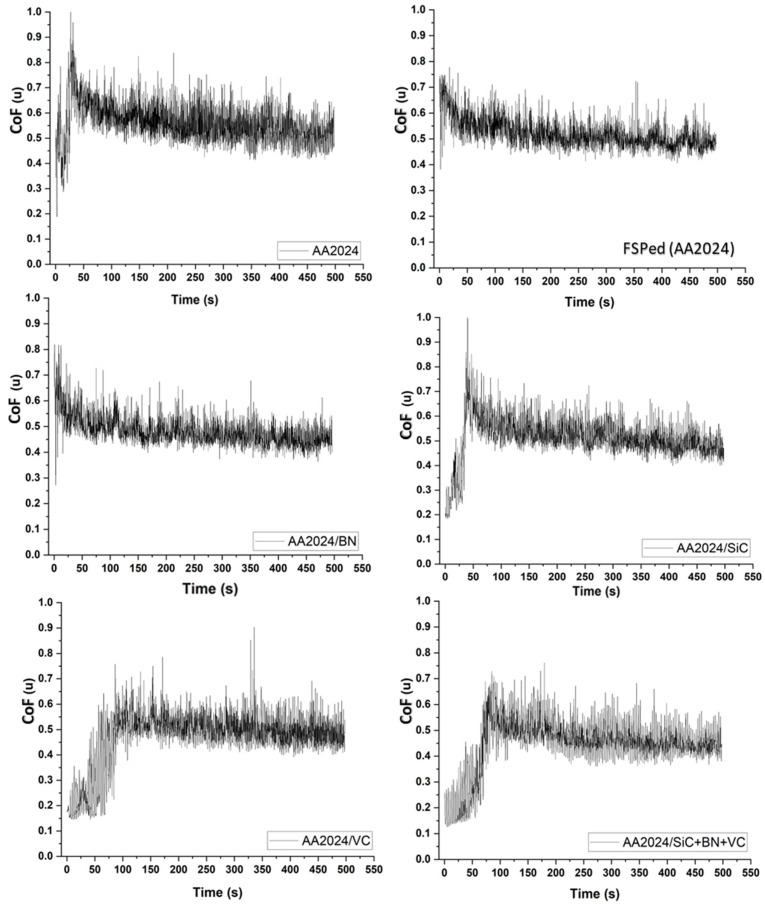
The coefficient of friction (CoF.) behavior during the 4 m traveling distance of the tested samples.

**Figure 13:**
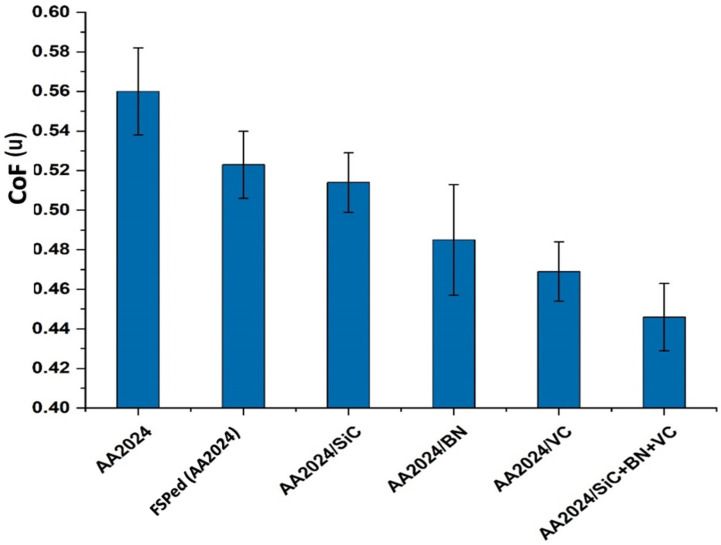
The relationship between the investigated samples and the mean value of the friction coefficient for the base, friction stir processing of wrought alloy, mono-composite, and hybrid composites samples.

The impact of reinforcement on the friction coefficient of the surface composites that were produced is demonstrated in Figure 12 and Figure 13. The experimental results obtained from AA2024/BN and AA2024/VC indicate that mono-composites exhibit a relatively low coefficient of friction compared to the tested samples, except for the hybrid composite. Including vanadium carbide reinforcement particles has significantly impacted the coefficient of friction of aluminum-based surface composites. As mentioned earlier, the phenomenon can be explained by the high microhardness and commendable wear resistance of vanadium carbide particles, which effectively mitigate the likelihood of plastic deformation of the composite surface upon experiencing frictional contact. Furthermore, adding vanadium carbide particles to the composite material resulted in a lower friction coefficient and wear rate. This is consistent with the findings of the previous investigation, and it suggests that vanadium carbide particles can be a promising way to improve the tribological properties of composite materials [42]. In addition, incorporating vanadium carbide particles enhances the stability of the friction interface through the reduction of adhesion between the surfaces.

Furthermore, the tribological characteristics of composites based on aluminum can be enhanced by incorporating reinforcement particles into the composite matrix. The utilization of boron nitride (BN) as a reinforcement particle has been recognized as a viable approach to augment the characteristics of composites based on aluminum. Numerous research studies have demonstrated that incorporating boron nitride reinforcement particles into the composite matrix leads to a noteworthy decrease in the coefficient of friction (COF). The decrease in the coefficient of friction (COF) is ascribed to the distinctive tribological characteristics of boron nitride, including its elevated thermal stability, reduced surface energy, and exceptional lubricating properties. Furthermore, including boron nitride reinforcement particles in the composite matrix enhances the composite’s microhardness and stiffness, thereby diminishing wear rates. Therefore the hybrid AA2024/SiC+BN+VC composite matrix shows excellent wear and low friction coefficient results.

**Figure 14:**
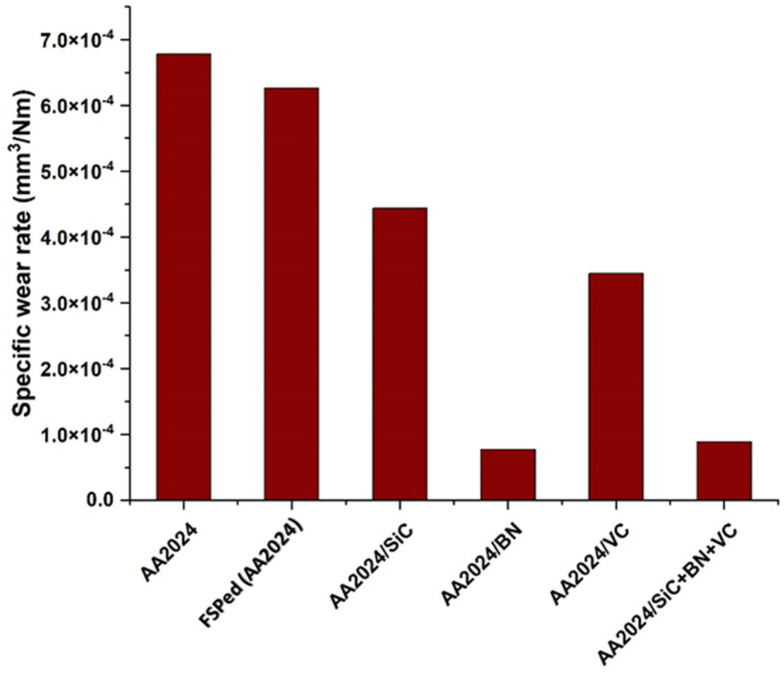
The specific wear rate of the investigated samples.

The quantification of volume loss was derived by utilizing the optical 3D profilometer to obtain the area of the worn surface, which was subsequently multiplied by the total distance traveled during the test. The findings indicate that the incorporation of BN, VC, and SiC particles resulted in a notable reduction in the specific wear rate of the surface composite matrix, as illustrated in Figure 14. Additionally, it was noted that the inclusion of BN particles resulted in a notable reduction in the specific wear rate. Nonetheless, its impact on the behavior of microhardness may be negligible. Despite this, due to their elevated microhardness and favorable lubrication properties, research has demonstrated that BN particles effectively reduce the specific wear rate. Research findings suggest that the reduction in the specific wear rate of surface composite matrices is positively correlated with the concentration of BN particles, wherein higher concentrations lead to a more significant decrease. Moreover, it has been demonstrated that the existence of BN particles aids in reducing the coefficient of friction between the two surfaces that are in contact and sliding against each other. The wear resistance of aluminum composites is significantly improved when both a hard reinforcement and soft reinforcement are added, these results are consistent with [43].

## 4. Conclusions

The present study has effectively produced single and hybrid composite matrices by utilizing the friction stir processing (FSP) technique.

The present summary provides a comprehensive analysis of the influence of ceramic nanoparticles, such as BN, VC, and SiC, on the microstructure of grain refinement, microhardness, and wear behavior of a composite based on an AA2024 alloy.

- The findings of the study showed that the addition of both singular and multiple nanoparticles of BN, VC, and SiC significantly influenced the microstructure grains. The inclusion of ceramic particles played a pivotal role in achieving a reduction in particulate size during friction stir processing. The mobile dislocations were pinned by the particles, which limited grain growth. The high surface-area-to-volume ratio of the particles facilitated their efficient activation within the material during processing, thereby contributing to the enhancement of the microstructure. As a result, the particle refinement level was 13.6 times greater than that of the wrought alloy.
- The uniform dispersion of ceramic nanoparticles is essential for improving metal matrix composites’ mechanical and physical properties (MMCs). In fact, the microhardness of hybrid composite matrices has been shown to increase by more than 95% when ceramic nanoparticles are uniformly dispersed.
- Hybrid composites exhibit a greater surface microhardness when compared to composites consisting of a single type of particle. This observed phenomenon can be ascribed to reinforcing particles, which seem to strengthen the interfaces between grains and impede the movement of dislocations within the composite material. The enhancement of internal grain boundaries and the initiation of a dispersion–strengthening mechanism are ascribed to the formation of uniformly distributed particles within the matrix. The hybrid composite surface demonstrated a significant improvement of 30% in tribological performance compared to the base metal.

## Figures and Tables

**Table 1:** The grade and chemical purity of the reinforcement particles.

	h-BN	VC	SiC
Chemical Purity	99.9%	99.9%	99.9%

**Table 2:** Chemical composition of the wrought alloy AA2024 (wt.%).

	Cu	Fe	Si	Mn	Mg	Zn	Cr	Al
AA2024	4.55	0.12	0.11	0.45	1.5	0.15	0.01	Remain

**Table 3:** Chemical composition of the tool steel K110 (wt.%).

	C	Si	Mn	Cr	Mo	V
K110	1.55	0.30	0.30	11.30	0.75	0.75

**Table 4:** The theoretical densities and volumes of the manufactured composites.

Composite	$\rho_{p}$, g/cm^3^	$V_{p}$, cm^3^	$\rho_{M}$ g/cm^3^	$V_{m}$ cm^3^	$V_{c}$ cm^3^	$\rho_{c}$ g/cm^3^
AA2024/SiC	3.21	2.49	2.78	3.1	4.5	2.96
AA2024/BN	2.1	2.49	2.78	3.1	4.5	2.49
AA2024/VC	5.77	2.49	2.78	3.1	4.5	4.05
AA2024/SiC+BN+VC	5.5	2.49	2.78	3.1	4.5	6.31

## Data Availability

The data is available on reasonable request from the corresponding author.
